# Supplementary material for: Screening and treatment of familial hypercholesterolemia in a French sample of ambulatory care patients: A retrospective longitudinal cohort study
Source: PLoS One. 2021 Aug 2;16(8):e0255345. doi: 10.1371/journal.pone.0255345 (PMC8328334; doi:10.1371/journal.pone.0255345)
Supplement: S1 Table — LLT, Lipid-lowering therapy; FH, Familial hypercholesterolemia. aData are presented as n (%). (DOCX) [file pone.0255345.s001.docx]

**S1 Table. LLT prescriptions of patients with definite or probable FH, at baseline and at month-6 of follow-up^a^.**

|  | **No LLT (n=43)** | **Statin (n=25)** | **Ezetimibe (n=1)** | **Ezetimibe + Statins (n=11)** | **Ezetimibe + Other LLT (n=4)** | **Ezetimibe + Other LLT + Statin (n=0)** | **Other LLT (n=6)** | **Other LLT + Statins (n=0)** | **Not reached follow-up (n=26)** | **Total (n=116)** | |
| --- | --- | --- | --- | --- | --- | --- | --- | --- | --- | --- | --- |
| **Baseline LLT prescription** | | | | | | | | | | |  |
| **No LLT** | 23 (71.9) | 1 (3.1) | - | - | - | - | - | - | 8 (25.0) | 32 (100) | |
| **Monotherapy** |  |  |  |  |  |  |  |  |  |  | |
| Statins | 10 (22.7) | 21 (47.7) | - | 1 (2.3) | 1 (2.3) | - | - | - | 11 (25.0) | 44 (100) | |
| Ezetimibe | 6 (85.7) | - | 1 (14.3) | - | - | - | - | - | - | 7 (100) | |
| **Combined therapy** |  |  |  |  |  |  |  |  |  |  | |
| Ezetimibe + Statins | 1 (5.6) | 1 (5.6) | - | 10 (55.6) | 1 (5.6) | - | 1 (5.6) | - | 4 (22.2) | 18 (100) | |
| Ezetimibe + Other LLT | 2 (100) | - | - | - | - | - | - | - | - | 2 (100) | |
| Ezetimibe + Other LLT + Statins | - | - | - | - | 1 (33.3) | - | 1 (33.3) | - | 1 (33.3) | 3 (100) | |
| Other LLT | 1 (14.3) | 1 (14.3) | - | - | 1 (14.3) | - | 3 (42.9) | - | 1 (14.3) | 7 (100) | |
| Other LLT + Statins | - | 1 (33.3) | - | - | - | - | 1 (33.3) | - | 1 (33.3) | 3 (100) | |

LLT, Lipid-lowering therapy; FH, Familial hypercholesterolemia

^a^Data are presented as n (%)
