# Supplementary material for: Screening and treatment of familial hypercholesterolemia in a French sample of ambulatory care patients: A retrospective longitudinal cohort study
Source: PLoS One. 2021 Aug 2;16(8):e0255345. doi: 10.1371/journal.pone.0255345 (PMC8328334; doi:10.1371/journal.pone.0255345)
Supplement: S2 Table — aData are presented as n (%). bAny statin, excluding statin + ezetimibe fixed combination. FH, Familial hypercholesterolemia. (DOCX) [file pone.0255345.s002.docx]

**S2 Table. Statin intensity of patients with definite or probable FH, at baseline and at month-6 of follow-up^a^.**

|  | **High** | **Moderate** | **Low** | **Not reached follow-up** | **Unknown** | **Total** |
| --- | --- | --- | --- | --- | --- | --- |
| **Statin intensity^b^ at baseline** | | | | | | |
| High | 7 (53.8) | - | - | 4 (30.8) | 2 (15.4) | 13 (100) |
| Moderate | 1 (2.9) | 15 (42.9) | - | 5 (14.3) | 14 (40.0) | 35 (100) |
| Low | - | 1 (10.0) | 3 (30.0) | 4 (40.0) | 2 (20.0) | 10 (100) |
| Missing | - | - | - | - | - | - |
| N/A | - | - | - | 4 (40.0) | 6 (60.0) | 10 (100) |

^a^Data are presented as n (%)

^b^Any statin, excluding statin + ezetimibe fixed combination

FH, Familial hypercholesterolemia
