# Supplementary material for: Screening and treatment of familial hypercholesterolemia in a French sample of ambulatory care patients: A retrospective longitudinal cohort study
Source: PLoS One. 2021 Aug 2;16(8):e0255345. doi: 10.1371/journal.pone.0255345 (PMC8328334; doi:10.1371/journal.pone.0255345)
Supplement: S3 Table — FH, Familial hypercholesterolemia; SD, Standard deviation; CI, Confidence interval; CVD, Cardiovascular disease; CIHD, Chronic ischemic heart disease; LDL, Low-density lipoprotein; Min, Minimum; Max, Maximum; Q, Quartile; LLT, Lipid lowering therapy. aThe index date is the date of the first entry of the DLCN score of the patient by the consented physician into the database. bAny statin, excluding statin + ezetimibe fixed combination. (DOCX) [file pone.0255345.s003.docx]

**S3 Table. Demographic and clinical characteristics and comorbidities of patients with definite, probable, possible or unlikely FH, at baseline^a^.**

|  | **Definite or Probable FH** | **Possible or Unlikely FH** |
| --- | --- | --- |
|  | **N = 116, % [95% CI]** | **N = 982, % [95% CI]** |
| **Demographic Characteristics** |  |  |
| Age, years, mean (SD) | 57.8 (14.0) | 61.5 (12.6) |
| Gender |  |  |
| Females | 65, 56.0 [46.5 - 65.2] | 623, 63.4 [60.3 - 66.5] |
| Males | 51, 44.0 [34.8 - 53.5] | 359, 36.6 [33.5 - 39.7] |
| **History of familial dyslipidaemia** | 65, 56.0 [46.5 - 65.2] | 416, 42.4 [39.2 - 45.5] |
| **Comorbidities** |  |  |
| Diabetes | 10, 8.6 [4.2 - 15.3] | 96, 9.8 [8.0 - 11.8] |
| Hypertension | 33, 28.4 [20.5 - 37.6] | 315, 32.1 [29.2 - 35.1] |
| CVD | 11, 9.5 [4.8 - 16.3] | 74, 7.5 [6.0 - 9.4] |
| Myocardial Infarction | - | - |
| Ischaemic Stroke | - | 4, 0.4 [0.1 - 1.0] |
| Peripheral Artery Disease | 2, 1.7 [0.2 - 6.1] | 18, 1.8 [1.1 - 2.9] |
| CIHD | 4, 3.4 [0.9 - 8.6] | 11, 1.1 [0.6 - 2.0] |
| Stable Angina | 3, 2.6 [0.5 - 7.4] | 16, 1.6 [0.9 - 2.6] |
| Unstable Angina | - | 3, 0.3 [0.1 - 0.9] |
| Carotid Artery Disease | 2, 1.7 [0.2 - 6.1] | 7, 0.7 [0.3 - 1.5] |
| Transient Ischemic Attack | 2, 1.7 [0.2 - 6.1] | 25, 2.5 [1.7 - 3.7] |
| Abdominal Aortic Aneurysm | 1, 0.9 [<0.1 - 4.7] | 3, 0.3 [0.1 - 0.9] |
| Obesity |  |  |
| Missing | 32, 27.6 [19.7 - 36.7] | 294, 29.9 [27.1 - 32.9] |
| Non-obese | 71, 61.2 [51.7 - 70.1] | 574, 58.5 [55.3 - 61.6] |
| Obese | 13, 11.2 [6.1 - 18.4] | 114, 11.6 [9.7 - 13.8] |
| **Clinical Characteristics** |  |  |
| LDL cholesterol level (mg/dL) |  |  |
| <160 | 12, 10.3 [5.5 - 17.4] | 185, 18.8 [16.4 - 21.4] |
| 160 to 190 | 8, 6.9 [3.0 - 13.1] | 100, 10.2 [8.4 - 12.2] |
| 190 to 250 | 65, 56.0 [46.5 - 65.2] | 630, 64.2 [61.1 - 67.2] |
| 250 to 325 | 19, 16.4 [10.2 - 24.4] | 23, 2.3 [1.5 - 3.5] |
| >325 | 6, 5.2 [1.9 - 10.9] | 5, 0.5 [0.2 - 1.2] |
| Missing | 6, 5.2 [1.9 - 10.9] | 39, 4.0 [2.8 - 5.4] |
| Number of days to index-date^a^ | Min: 0; Q1: 5; Q2: 43; Q3: 287; Max: 726 | Min: 0; Q1: 6; Q2: 60; Q3: 210; Max: 1,077 |
| Total cholesterol level (mg/dL) |  |  |
| <200 | 8, 6.9 [3.0 - 13.1] | 79, 8.0 [6.4 - 9.9] |
| ≥200 | 96, 82.8 [74.6 - 89.1] | 819, 83.4 [80.9 - 85.7] |
| Missing | 12, 10.3 [5.5 - 17.4] | 84, 8.6 [6.9 - 10.5] |
| Number of days to index-date^a^ | Min: 0; Q1: 5.5; Q2: 43; Q3: 265.5; Max: 726 | Min: 0; Q1: 6; Q2: 61; Q3: 213; Max: 1,077 |
| Triglyceride level (mg/dL) |  |  |
| <150 mg/dL | 61, 52.6 [43.1 - 61.9] | 597, 60.8 [57.7 - 63.9] |
| 150 to 200 | 23, 19.8 [13.0 - 28.3] | 156, 15.9 [13.7 - 18.3] |
| 200 to 500 | 20, 17.2 [10.9 - 25.4] | 158, 16.1 [13.8 - 18.5] |
| ≥500 | - | 2, 0.2 [<0.1 - 0.7] |
| Missing | 12, 10.3 [5.5 - 17.4] | 69, 7.0 [5.5 - 8.8] |
| Number of days to index-date^a^ | Min: 0; Q1: 6; Q2: 47.5; Q3: 310.5; Max: 726 | Min: 0; Q1: 6; Q2: 62; Q3: 209; Max: 1,077 |
| **LLT prescription** |  |  |
| No LLT | 32, 27.6 [19.7 - 36.7] | 422, 43.0 [39.9 - 46.1] |
| Any LLT prescription | 84, 72.4 [63.3 - 80.3] | 560, 57.0 [53.9 - 60.1] |
| **Monotherapy** |  |  |
| Ezetimibe | 7, 6.0 [2.5 - 12.0] | 9, 0.9 [0.4 - 1.7] |
| Statins | 44, 37.9 [29.1 - 47.4] | 417, 42.5 [39.3 - 45.6] |
| **Combined therapy** |  |  |
| Ezetimibe + Statins | 18, 15.5 [9.5 - 23.4] | 60, 6.1 [4.7 - 7.8] |
| Ezetimibe + Other LLTs | 2, 1.7 [0.2 - 6.1] | - |
| Ezetimibe + Other LLTs + Statins | 3, 2.6 [0.5 - 7.4] | 5, 0.5 [0.2 - 1.2] |
| Other LLTs | 7, 6.0 [2.5 - 12.0] | 54, 5.5 [4.2 - 7.1] |
| Other LLTs + Statins | 3, 2.6 [0.5 - 7.4] | 15, 1.5 [0.9 - 2.5] |
| **Statin intensity^b^** |  |  |
| Unknown | 10, 8.6 [4.2 - 15.3] | 18, 1.8 [1.1 - 2.9] |
| Low | 10, 8.6 [4.2 - 15.3] | 135, 13.7 [11.7 - 16.1] |
| Moderate | 35, 30.2 [22.0 - 39.4] | 310, 31.6 [28.7 - 34.6] |
| High | 13, 11.2 [6.1 - 18.4] | 34, 3.5 [2.4 - 4.8] |

FH, Familial hypercholesterolemia; SD, Standard deviation; CI, Confidence interval; CVD, Cardiovascular disease; CIHD, Chronic ischemic heart disease; LDL, Low-density lipoprotein; Min, Minimum; Max, Maximum; Q, Quartile; LLT, Lipid lowering therapy

^a^The index date is the date of the first entry of the DLCN score of the patient by the consented physician into the database

^b^Any statin, excluding statin + ezetimibe fixed combination.
